# Integrating drones in response to public health emergencies: A combined framework to explore technology acceptance

**DOI:** 10.3389/fpubh.2022.1019626

**Published:** 2022-10-28

**Authors:** Stav Shapira, Jessica R. Cauchard

**Affiliations:** ^1^School of Public Health, Faculty of Health Sciences, Ben-Gurion University of the Negev, Beer Sheva, Israel; ^2^Magic Lab, Department of Industrial Engineering and Management, Faculty of Engineering, Ben-Gurion University of the Negev, Beer Sheva, Israel

**Keywords:** drone, public health emergencies, TAM, TTF, risk perception, social motivation

## Abstract

The aim of the study was to propose and test an integrated model combining the technology acceptance model (TAM), task-technology fit (TTF), social motivation, and drone-related perceived risks to explore the intention to use drones in public health emergencies (PHEs). We conducted a survey among the Israeli population, yielding a sample of 568 participants. Structural equation modeling was implemented to test the research hypotheses. The results showed that our integrated model provided a robust and comprehensive framework to perform an in-depth investigation of the factors and mechanisms affecting drone acceptance in PHEs. First, ease of use, attitudes, individual-technology fit, task-technology fit, and social influence significantly and directly influenced users' behavioral intention to utilize drone technology. Second, attitudes were significant mediators of the effects of social influence and perceived risks on the intention to use drones. Finally, significant relationships between TAM, TTF, social motivation, and perceived risks were also observed. Theoretical aspects and practical implications—which can serve as the basis for shaping a positive development in drone public acceptance in PHEs and in general—are discussed.

## Introduction

The COVID-19 pandemic that erupted in December 2019 led to the emergence of various technologies worldwide for tackling the virus spread and its health impacts, as well the social aspects of the pandemic (1). These technologies included, among others, geospatial technology, artificial intelligence, big data, telemedicine, smart applications, and robotics (2). Within this context, much attention has been given to drones (also known as unmanned aerial vehicles). Drones are a type of flying robot, the specific attributes of which enable performing various actions in a remote manner: an important feature during a viral pandemic. Drone applications during the COVID-19 pandemic have been varied and include monitoring, surveillance, delivery, and increasing awareness (3). The potential benefits of drone use in responding to public health emergencies (PHEs) such as COVID-19 are well documented; however, the technical, security, and privacy risks when using drones in this context have also been highlighted (4). These risks combined with the fact that drones are considered a relatively innovative technology pose a challenge in shaping positive development in drone public acceptance: a key to the increase of drone use. In this study we present a comprehensive framework to investigate drone acceptance in the specific context of a PHE, by combining two theoretical models for technology acceptance and utilization—the technology acceptance model (TAM) and the task-technology fit theory (TTF)—as well as the addition of context variables. The theoretical background provides a short overview of drone technology, its applications during PHEs with a specific focus on the COVID-19 pandemic, as well as related challenges and risks. Next, we describe the recent insights related to drone acceptance research and provide theoretical and empirical justification for the use of TAM and TTF in the specific context of drone use in PHEs. In the remainder of the paper we present the research methods used for the current analysis (including the research model and detail the process of hypothesis development), and the main findings. In the final section, we present a discussion of the study's implications as well as the conclusions, limitations, and directions for future research. We conducted a thorough literature review to gain a comprehensive understanding of the investigated issue. In the following section we summarize the literature relevant to the integration of drones in civil usage and specifically in PHEs, to drone acceptance research, and to the justification of using TAM and TTF in this specific domain.

### Theoretical background

Drone technology usage has increased dramatically in recent years within a wide range of disciplines. Drones have multiple applications such as providing real-time data, offering aerial mailing and delivery solutions, and performing surveillance tasks (5). All of these make drones a useful and in-demand technology in both civic domains such as agriculture (6), commercial industry (7), research (8), medicine and healthcare (9), disaster management (10), law enforcement (11), as well as in military use (12).

Notwithstanding the above, the use of drone technology is known to pose societal challenges and risks related to citizens' safety, security, and privacy (13). The concerns that have been raised in these regards range from safety issues related to technical malfunctions that may lead to property damage or injury (14, 15); security issues related to intentional misuse of the technology such as for terrorism purposes, delivering illicit goods, or delivery theft (16); and privacy issues that stem from unintentional service disruptions (e.g., delivery to wrong address) or from intentional privacy invasion (13). In order to minimize these potential risks, countries worldwide have established legal frameworks and design standards (17), and additional technological improvements related to flight duration, reliability, and ease of use are constantly introduced (18) in an effort to foster drone applicability and acceptance.

The recent outbreak of the COVID-19 pandemic once again provoked discussion regarding drone application and integration in response efforts to large-scale PHEs. A large number of studies have previously identified the benefits inherent in incorporating drones in PHE management, both for (a) short-term events such as natural disasters (19), in which the main advantages recognized have been related to search and rescue missions and to delivering humanitarian aid under extreme conditions of collapsed healthcare and transportation infrastructures; and (b) for more prolonged situations, the most recent example of which is, of course, the ongoing global COVID-19 pandemic. Drones have assisted countries in different ways to reduce the spread of the virus through disinfection and screening infection symptoms (20), monitoring physical distancing during lockdowns (21), delivering medical supplies (22), and increasing public awareness (23, 24). All of the above are performed without any person-to-person contact, reducing the risk of infection.

Notwithstanding the foregoing, other voices have posed critical questions and emphasized current challenges regarding various technical, practical, and ethical issues of developing and deploying drones for the purpose of addressing COVID-19 (25). For example, a recent study that investigated a drone-based system for combating the COVID-19 pandemic stressed, among other things, the technical challenges such as collision avoidance, the ability of drones to operate in internal spaces (e.g., private homes) without causing disruptions, and other infrastructure constraints related to collecting data and providing services on a large scale (4). The main ethical argument in regard to drone application during COVID-19 has been the lack of sufficient consideration given to the consequences for civil liberties, mainly the right to privacy (26). Another concern has been the possibility of psychological resistance among members of the public as a result of the ways in which drones might be used during the pandemic (27, 28). In this regard, it should be noted that the deployment of drone-based solutions in the global north might be quite different from the global south, due to the different political and socioeconomic features (26). In a recent report by UNICEF on how drones can be used to combat COVID-19, it was recommended that local sensitization of communities and stakeholders be performed before and during drone program implementation, in order to raise public awareness about this technology, ultimately facilitating its acceptance (25). As such, there is a clear need to conduct research that examines the application and acceptance of this technology among different populations worldwide and to chart how individuals engage with the benefits, risks, and uses of drones in the context of a PHE.

Public acceptance is key to the increase of drone use, both during routine and emergency times (29). In general, it can be argued that although drone technology is not that new, most of the world's population is still in a formative phase where attitudes and perceptions toward the acceptability of drones are developing and taking shape (16, 30). Previous research indicated that public willingness to accept drones depended on the context in which the technology was being used (31). For example, people's acceptance of drones was found to be higher in industrial areas than in residential areas (30) and in situations perceived as severe such as earthquakes or terror attacks than in more daily/routine situations (1). As such, one could speculate that people may be more receptive toward drones offering medical assistance during emergencies than with drones performing other tasks during ordinary/routine times.

Several studies that explored drone acceptance utilized the knowledge, attitude, and practice (KAP) model: a widely used approach for studying human behavior based on the principle that increasing knowledge will result in changing attitudes and practices (32). A US-based study applying this approach found that knowledge about potential uses, perceived risks, and perceived benefits all had a strong impact on drone acceptance (33). Conversely, another US-based KAP study reported that knowledge about drones and their uses did not alter the public's perception of them, and the researcher concluded that this finding was related to the high impact of risks and concerns related to drones (34). These latter notions were supported by another study from Singapore that utilized a KAP survey and found that perceived benefits of and concerns about drones were responsible for varying levels of acceptance across different contexts (30). The KAP model has some major weaknesses. First, it assumes a direct relationship between knowledge and action, a notion that has gained criticism as well as empirical evidence that refutes it (35, 36). Second, it does not capture other external factors that may be involved in the acceptance of new technologies (37). These factors include, among others, social motivation, which has been found to have a positive influence on attitudes which, in turn, affect behavioral intentions in the context of drone acceptance (38).

In light of the above, we chose to suggest a comprehensive research framework that includes two of the most frequently employed models for exploring new technology acceptance and utilization: the technology acceptance model (TAM) for adoption and the task-fit technology theory (TTF) for utility. Additional dimensions of social motivation and drone-related risks were also incorporated. Previous studies have utilized this extended framework to explore the intention to use various technological innovations (39–41), and others have highlighted its potential benefits in exploring drone acceptance (42).

The technology acceptance model (TAM) is considered the most established and frequently used model for investigating factors affecting users' acceptance of technologies (43). The TAM is derived from theories in the fields of psychology and sociology such as the theory of reasonable action (TRA) and the theory of planned behavior (TPB), which both explain and predict individual behavior (44). The TAM suggests that the user's intention to use a certain technology can be explained *via* three main constructs: *perceived ease of use, perceived usefulness*, and *attitude toward using*. According to the model, the *attitude* of an individual toward a specific technology is a major determinant of whether the user will actually use or reject it. The *attitudes* of the user are considered to be influenced by two major beliefs: *perceived usefulness* and *perceived ease of use*, with *perceived ease of use* having a direct influence on *perceived usefulness* (45). The TAM has been applied in various technological contexts [e.g., (40, 46)]. Nonetheless, the original model has been criticized for not referring to factors such as social influence and motivation (43, 44), which have been documented to be significant antecedents in the process of technology acceptance (40, 47). For the specific context of drones, studies have highlighted the significant role of various social factors in shaping users' perceptions and their potential ability to alter technology acceptance (16, 48, 49). Therefore, we included variables such as social influence, social recognition, and specific drone-related risks to gain a better understanding of the facilitators and inhibitors involved in drone acceptance. In addition, the fit between task and technology, which is absent from the TAM but is the focus of the TTF, is considered an important factor in long-term technology utilization. Therefore, a model combining the TAM and TTF can provide a better explanation for the variance in user acceptance of technological innovations (40, 41).

The TTF model focuses on the fit between tasks and the technologies that are designed to support people in the performance of them. Both task and technology characteristics can impact this fit, which consequently determines users' performance and utilization. The TTF has been applied to a wide range of technological contexts (50) and has also been suggested as an important lens through which drone acceptance and utilization should be viewed (42). However, very little is known about whether a good task-technology fit would affect drone public acceptance, and even less is known in the specific context of a PHE. Like the TAM, the TTF also does not address social factors, potentially limiting its ability to predict adoption of technology applied in both private and public spaces (such as drones). Thus, integrating social factors such as those described above may help to bypass this limitation.

## Materials and methods

**Figure 1:**
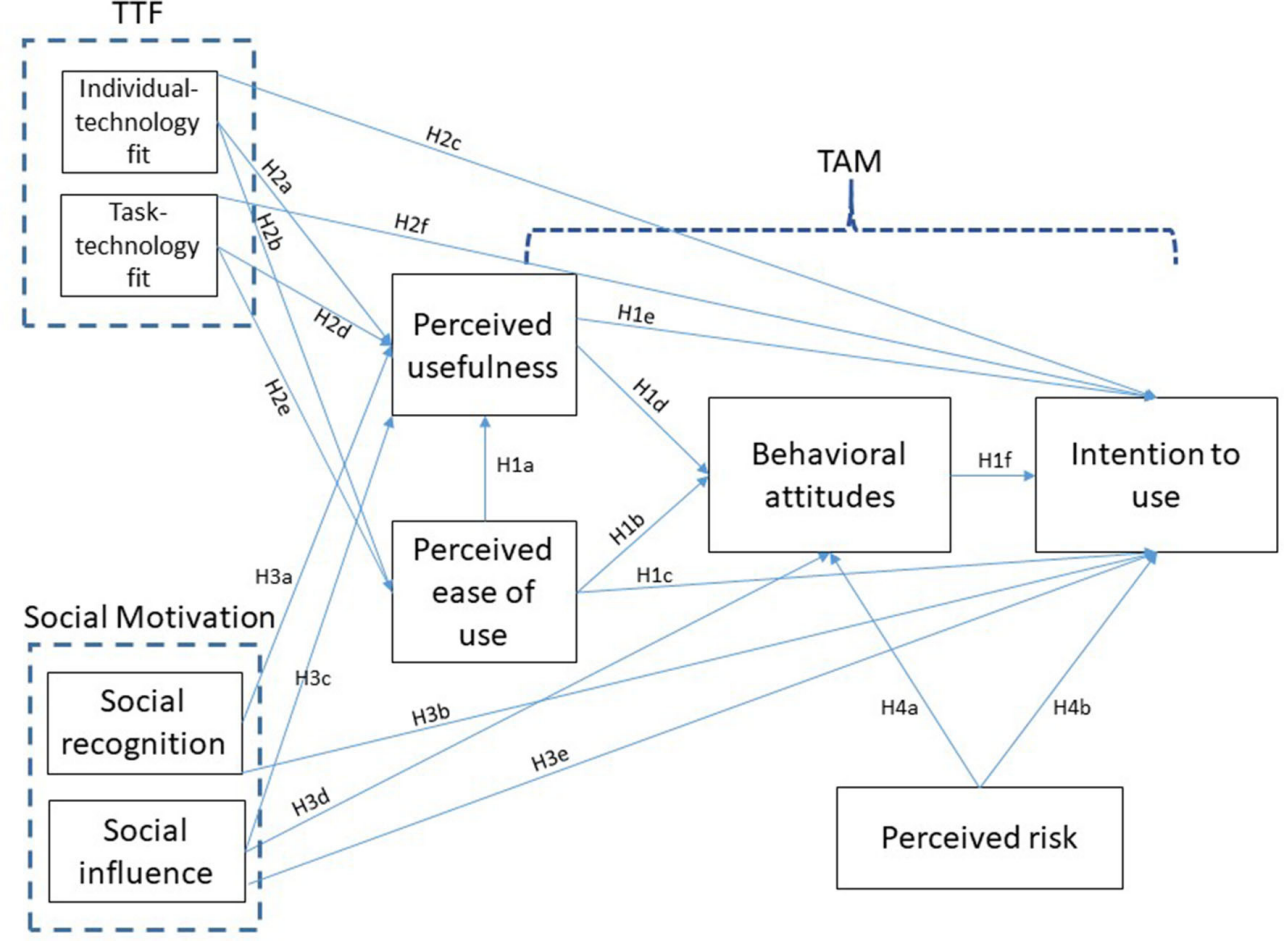
Proposed research model.

Drawing upon the theoretical background of the extended TAM, the TTF, and social motivations, we have proposed a research model that aims to identify several factors as predictors of intention to use drones in the context of a PHE. The interplay between these factors is depicted in the conceptual model displayed in Figure 1.

The premise here is that intention to use drones is jointly determined by perceived usefulness and attitudes, which are functions of perceived ease of use, TTF, perceived risks, and social motivations. Furthermore, we hypothesized that TTF, perceived risks, and social motivations might also have direct associations with the intention to use drones.

Perceived ease of use can be defined as the extent to which a person believes that using a new technology will be effort-free: for example, the ease of learning to operate or communicate with a drone. Previous studies have shown that perceived ease of use has a positive effect on perceived usefulness in the context of human-robot cooperation (51) and on attitudes toward using drones (28). In addition, perceived ease of use was highlighted as an important factor for drone utilization (5), and it is thus possible to assume that this construct could directly or indirectly affect the intention to use drones. We therefore proposed the following research hypotheses:

**H1a**: Perceived ease of use would have a positive effect on the perceived usefulness of drones during a PHE.
**H1b**: Perceived ease of use would have a positive effect on attitudes toward using drones during a PHE.
**H1c**: Perceived ease of use would have a positive effect on intention to use drones during a PHE.

Perceived usefulness is defined as the extent to which a person believes that using a particular technology will enhance their job performance (45). In the context of a PHE this notion may be related to the perceived ability of a drone to assist in maintaining health and wellbeing. Perceived usefulness has been shown to affect both attitudes and intention to use drones for online order delivery during COVID-19 (28). Thus, we expected to see such findings replicated in the context of performing different tasks related to the maintenance of health during the pandemic:

**H1d**: Perceived usefulness would have a positive effect on attitudes toward using drones during a PHE.
**H1e**: Perceived usefulness would have a positive effect on intention to use drones during a PHE.

Attitudes toward using drones in a PHE refers to the extent to which a person develops positive or negative feelings related to the drone in this specific context: for example, the belief that incorporating drones into emergency response efforts is a good vs. bad idea. Behavioral attitudes toward drones were found to positively affect the intention to use drones for online order delivery during COVID-19 (28)—thus, we expected this finding to be replicated as well:

**H1f**: Positive attitudes toward using drones would have a positive effect on intention to use them during a PHE.

Previous studies have established a link between the TTF and TAM models through the effects that the task-technology fit factors have on the main constructs of the TAM: perceived ease of use and perceived usefulness (40, 41). Others demonstrated the direct effect of TTF on intention to use technology (52, 53). The choice to operate or interact with a drone during a PHE is intuitively influenced by two technology features: individual-technology fit (e.g., a person's belief that they can independently interact with a drone) as well as task-technology fit (e.g., the belief that using drones can assist in maintaining health). As no previous studies have provided empirical evidence for the association between TTF and TAM factors in the context of drone use, we proposed the following hypotheses:

**H2a**: Individual-technology fit would have a positive effect on the perceived usefulness of drones.
**H2b**: Individual-technology fit would have a positive effect on the perceived ease of use of drones.
**H2c**: Individual-technology fit would have a positive effect on intention to use drones.
**H2d**: Task-technology fit would have a positive effect on the perceived usefulness of drones.
**H2e**: Task-technology fit would have a positive effect on the perceived ease of use of drones.
**H2f**: Task-technology fit would have a positive effect on intention to use drones.

Drones are an emergent technology, and their uses are often conducted in public spaces. In the context of PHEs, drones will most likely operate in the service of health services or other national institutions. Thus, social motivation can be conceptualized as the positive recognition of drone-expected-benefits in healthcare and as the belief that other people should also accept and trust drones, factors that may play a central role in individual willingness to adopt and utilize this technology (54). Social recognition has been shown to influence the perceived usefulness of new technologies (40) as well as people's behavioral intentions (55). However, the implementation of social recognition in the context of drones has yet to be explored. Accordingly, we hypothesized as follows:

**H3a**: Social recognition would have a positive effect on the perceived usefulness of drones.
**H3b**: Social recognition would have a positive effect on intention to use drones.

An additional conceptualization of social motivation in the context of drones in PHEs may be related to social influence. Social influence has been extensively studied in the context of adopting new technologies. Several studies have demonstrated its significant role in impacting the perceived usefulness of new technologies (56); attitudes toward new technologies (57); and as a predictor of intention to use technologies such as home healthcare robots (58), and mobile payment (56). In the context of drones, social influence was investigated in regard to public perceptions of drone use for food delivery (59). However, a comprehensive investigation regarding its effect on other constructs involved in adoption processes has yet to be performed. Thus, we hypothesized as follows:

**H3c**: Social influence would have a positive effect on the perceived usefulness of drones.
**H3d**: Social influence would have a positive effect on the attitude toward using drones.
**H3e**: Social influence would have a positive effect on the intention to use drones.

Perceived risk is often used as an additional variable in the TAM (60–62). In the context of drone use in PHEs, we therefore explored two of the most common drone-related risks: performance risk (i.e., the possibility that the drone might physically harm someone while attempting to provide aid) and privacy risk (i.e., invasion of privacy by a flying drone and the potential loss of control over personal medical information) (13). Perceived risks have been found to negatively influence drone delivery adoption mainly through impacting attitudes (63), but some evidence also suggests a direct effect on intention to use with regard to other technologies (64). Accordingly, we hypothesized as follows:

**H4a**: Perceived risks about drones would have a negative effect on attitudes toward using drones.
**H4b**: Perceived risks about drones would have a negative effect on intention to use drones.

In this study we employed a cross-sectional design using a quantitative survey in order to test the hypotheses formulated in the previous sections. Questionnaire development and data collection are elaborated upon below.

### Questionnaire development

We used a questionnaire survey consisting of two sections. The first section included demographic information and an item that examined familiarity with drones (i.e., do you know what a drone is?). The second section featured questions measuring the nine constructs in the research model. Each of the constructs was measured by multiple-item, self-reported scales (apart from one construct—individual-technology fit/ITF—which was measured by a single item), as shown in Appendix A. All items were measured using a 5-point Likert scale, ranging from 1 (strongly disagree) to 5 (strongly agree), which is the most frequently used type of response to measure perceptions and attitudes (65).

### Participants and data collection

The data were collected in July 2020 by undertaking a population survey. A randomized sample of the Israeli adult population was engaged through an online polling service. We collaborated with iPanel (66), a survey company with the largest online panel in the country. It adheres to the rigorous standards of the European Society for Opinion and Marketing Research (ESOMAR). Only adults (≥18 years) were eligible to participate. The survey targeted a specific distribution of age and gender, broadly reflecting the population of Israel. Overall, 568 valid surveys were returned. The participants' demographics are depicted in Table 1.

**Table 1:** Demographic characteristics of the study participants^#^.

		***n* (%)**
Gender	Female	295 (52%)
	Male	269 (47.5%)
Age (years)	Mean ± SD	48.9 ± 20.3
	Min-Max (in years)	18–87
Educational level	Elementary	20 (3.5%)
	Secondary (high school)	143 (25.5%)
	Tertiary (vocational)	179 (32%)
	Tertiary (university or college)	216 (39%)

# Without missing values.

### Data analysis

Data analysis was performed in two steps. First, we examined the fitness and validity of the model constructs (67, 68) by assessing reliability (Cronbach's alpha); convergent validity (factor loadings, composite reliabilities/CRs, and average variance extracted/AVE, for each construct); and discriminant validity (squared root of AVE). Table 2 demonstrates item loadings, AVE, CRs and Cronbach's alpha values for all constructs in the measurement model, and Table 3 demonstrates the inter-correlation analysis and discriminant validity. The analyses were conducted using SPSS version 26. Next, we examined the structural model to investigate the strength and direction of the relationships among the theoretical constructs by performing Structural Equation Modeling (SEM) using the bootstrapping method. The main principle underlying the bootstrapping method is that it allows the researcher to simulate repeated subsamples from an original database, allowing the assessment of the stability of parameter estimates and reporting their values with a greater degree of accuracy (69, 70). We used bootstrapping based on 5,000 iterations to assess the estimators and 95% confidence intervals for direct and indirect effects. The following indices were used to evaluate the model: chi-square, which is acceptable when the value is not significant; the goodness of fit index (GFI); the comparative fit index (CFI); the non-normed fit index (NFI); and the root mean square error of approximation (RMSEA) (70–72). SEM was tested using AMOS software.

**Table 2:** Construct reliability and convergent validity.

**Construct**	**Construct code**	**Items loading**	**AVE**	**CRs**	**Cronbach's a**
PU	1	0.804	0.55	0.907	0.88
	2	0.803			
	3	0.792			
	4	0.752			
	5	0.729			
	6	0.710			
	7	0.683			
	8	0.643			
PEOU	1	0.827	0.54	0.855	0.78
	2	0.769			
	3	0.723			
	4	0.710			
	5	0.642			
ATT	1	0.846	0.60	0.816	0.65
	2	0.822			
	3	0.638			
ITU	1	0.895	0.69	0.899	0.84
	2	0.881			
	3	0.827			
	4	0.710			
ITF	1	NA			
TTF	1	0.872	0.72	0.885	0.8
	2	0.838			
	3	0.835			
SR	1	0.930	0.86	0.928	0.84
	2	0.930			
SI	1	0.892	0.71	0.881	0.79
	2	0.820			
	3	0.818			
RISK	1	0.921	0.74	0.895	0.82
	2	0.888			
	3	0.765			

NA = not applicable, as contains only 1 item.

**Table 3:** Inter-construct correlations and discriminant validity.

	**M (SD)**	**PU**	**PEOU**	**ATT**	**ITU**	**ITF**	**TTF**	**SR**	**SI**	**RISK**
PU	3.8 (0.84)	**0.74**								
PEOU	3.5 (0.80)	0.312^**^	**0.74**							
ATT	4.1 (0.72)	0.462^**^	0.417^**^	**0.77**						
ITU	3.8 (0.78)	0.437^**^	0.491^**^	0.596^**^	**0.83**					
ITF	3.7 (0.92)	0.361^**^	0.577^**^	0.305^**^	0.527^**^	NA				
TTF	3.4 (0.85)	0.509^**^	0.399^**^	0.442^**^	0.618^**^	0.511^**^	**0.85**			
SR	3.5 (0.84)	0.437^**^	0.421^**^	0.508^**^	0.619^**^	0.441^**^	0.605^**^	**0.93**		
SI	2.8 (0.87)	0.189^**^	0.121^**^	0.100^*^	0.266^**^	0.226^**^	0.380^**^	0.425^**^	**0.84**	
RISK	2.8 (0.96)	−0.141^**^	−0.224^**^	−0.379^**^	−0.274^**^	−0.066	−0.218^**^	−0.291^**^	−0.064	**0.95**

Discriminant validity (squared root of average variance extracted) for each construct is presented in bold numbers.

* p < 0.05.

** p < 0.001.

NA = not applicable, as contains only 1 item.

## Results

In order to assess the hypothesized model, SEM was performed, using the bootstrapping method. To test the research hypotheses, TTF, social motivation, and risk aspects were exogenous variables; perceived usefulness, perceived ease of use, and behavioral attitudes served as mediators; and intention to use drones was the outcome tested. In addition, we controlled for the influence of age, gender, and education. The model showed good fit: chi-square/degree of freedom = 4.27, CFI = 0.99, GFI = 0.99, NFI = 0.99, TLI = 0.93, RMSEA = 0.08.

**Figure 2:**
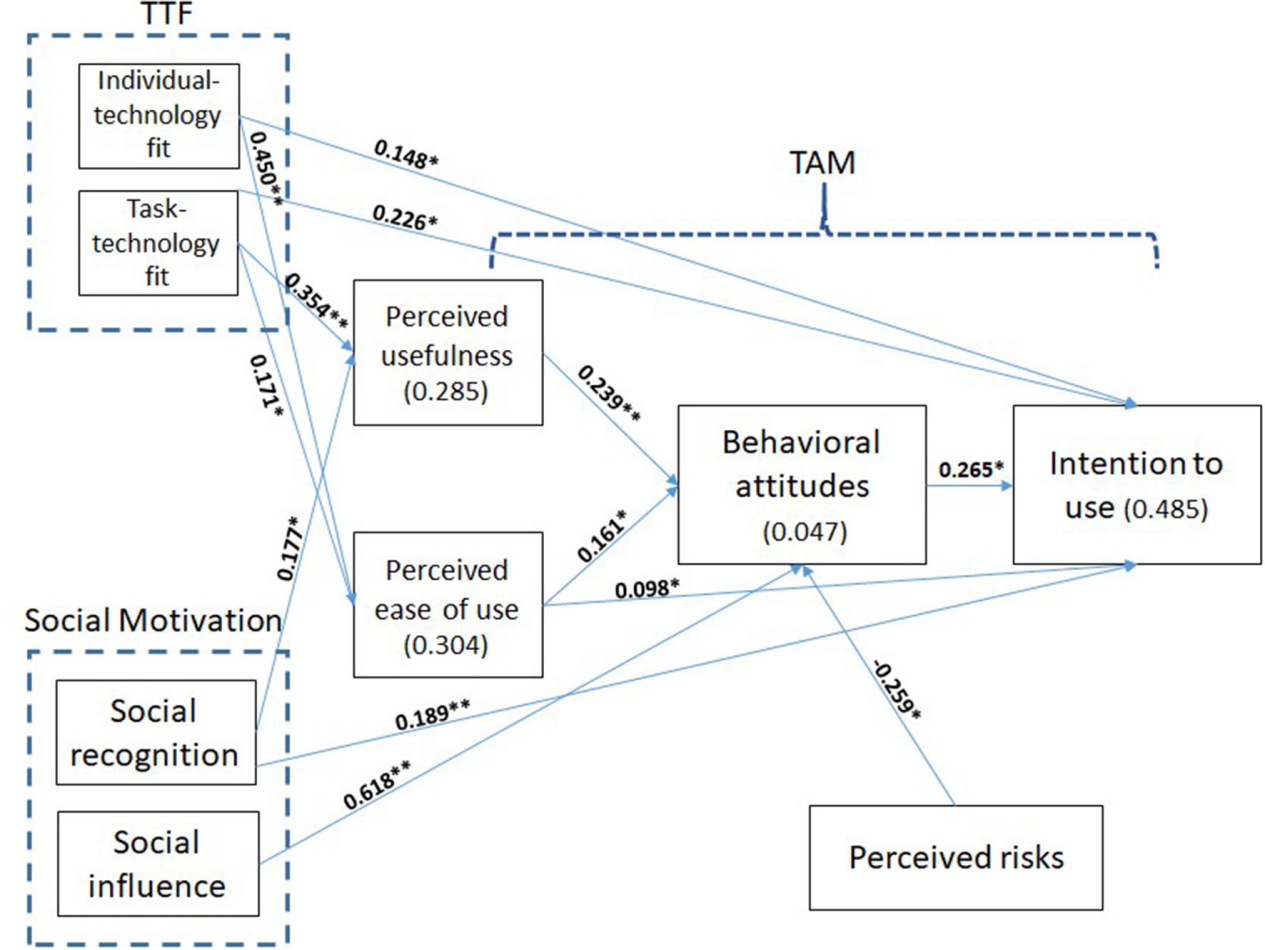
Path analysis. *p < 0.05; **p < 0.001.

Figure 2 illustrates the *R*^2^-values, which represent the amount of variance explained by the independent variables, and estimates of the path coefficients of the proposed research model, which indicate the strengths of the relationships between the dependent and independent variables. Perceived usefulness was found to be significantly determined by the two exogenous variables (i.e., task-technology fit and social recognition), resulting in an *R*^2^ of 0.248. Likewise, perceived ease of use was found to be significantly determined by the two exogenous variables (i.e., individual-technology fit and task-technology fit), resulting in an *R*^2^ of 0.304. Behavioral attitudes were significantly determined by perceived usefulness, perceived ease of use, social influence, and perceived risks, resulting in an *R*^2^ of 0.047. The dependent variable, intention to use drones, was significantly determined by perceived ease of use, behavioral attitudes, individual-technology fit, task-technology fit, and social recognition, resulting in an *R*^2^ of 0.485. Thus, the combined effect of these five variables explained 48.5% of the variance in intention to use drones. Table 4 summarizes the hypotheses testing results of the standardized path coefficients and path significances. Most of the paths were significant in the expected direction.

**Table 4:** Model path analysis.

**The hypotheses**	**Path coefficient**	***P-*** **value**		**Conclusion**
**Direct paths**				
H1a	Perceived ease of use → Perceived usefulness	0.069	0.104	Not supported
H1b	Perceived ease of use → Behavioral attitudes	0.161	0.014^*^	Supported
H1c	Perceived ease of use → Intention to use	0.098	0.019^*^	Supported
H1d	Perceived usefulness → Behavioral attitudes	0.239	0.006^**^	Supported
H1e	Perceived usefulness → Intention to use	0.007	0.924	Not supported
H1f	Behavioral attitudes → Intention to use	0.265	0.012^*^	Supported
H2a	Individual-technology fit → Perceived usefulness	0.070	0.089	Not supported
H2b	Individual-technology fit → Perceived ease of use	0.450	0.008^**^	Supported
H2c	Individual-technology fit → Intention to use	0.148	0.005^**^	Supported
H2d	Task-technology fit → Perceived usefulness	0.354	0.004^**^	Supported
H2e	Task-technology fit → Perceived ease of use	0.171	0.014^*^	Supported
H2f	Task-technology fit → Intention to use	0.226	0.012^*^	Supported
H3a	Social recognition → Perceived usefulness	0.177	0.018^*^	Supported
H3b	Social recognition → Intention to use	0.189	0.007^**^	Supported
H3c	Social influence → Perceived usefulness	−0.042	0.273	Not supported
H3d	Social influence → Behavioral attitudes	0.618	0.013^*^	Supported
H3e	Social influence → Intention to use	0.012	0.689	Not supported
H4a	Perceived risks → Behavioral attitudes	−0.259	0.012^*^	Supported
H4b	Perceived risks → Intention to use	0.005	0.915	Not supported
**Indirect paths**				
	Perceived usefulness → Behavioral attitudes → Intention to use	0.063	0.007^**^	
	Perceived ease of use → Intention to use^#^	0.048	0.016^*^	
	Individual-technology fit → Intention to use^#^	0.071	0.010^*^	
	Task-technology fit → Intention to use^#^	0.050	0.008^**^	
	Social recognition → Intention to use^#^	0.012	0.043^*^	
	Social influence → Intention to use^#^	0.161	0.011^*^	
	Perceived risks → Intention to use^#^	−0.069	0.007^**^	

# More than one path available.

* p < 0.05.

** p < 0.001.

### Relationship in TAM

Hypotheses 1a to 1f addressed the relationship in the TAM, which is related to perceived usefulness, perceived ease of use, behavioral attitudes, and intention to use drones, and all except Hypotheses 1a and 1e were supported. Although the direct link between perceived usefulness and intention was not significant, we did find that a significant indirect path existed between these variables, mediated by behavioral attitudes. In addition, another significant indirect path was found between perceived ease of use and intention, and was also mediated by behavioral attitudes.

### Relationship in TTF and TAM

Hypotheses 2a−2f assessed the relationship between the variables of TTF (individual-technology fit and task-technology fit) and the variables of TAM. We explored both the direct links between the TTF variables and intention to use drones (H2c and H2f), as well as the links with perceived usefulness (H2a and H2d) and perceived ease of use (H2b and H2e). All hypotheses apart from H2a were supported. In addition, significant indirect paths were found between individual-technology fit, task-technology fit, and intention to use, mediated by perceived usefulness, perceived ease of use, and behavioral attitudes.

### Relationship between social motivation and TAM

Hypotheses 3a−3e explored the relationship between variables of social motivation (social recognition and social influence) and TAM variables. We explored both the direct links between the social motivation variables and intention to use drones (H3b and H3e), as well as the links between social motivation and perceived usefulness (H3a and H3c), and the link between social influence and behavioral attitudes (H3d). All hypotheses were supported except for H3c and H3e. In addition, significant indirect paths between social motivation variables and intention to use were detected, mediated by perceived usefulness and behavioral attitudes.

### Relationship between perceived risks and TAM

Hypotheses 4a and 4b addressed the relationship between drone-related risks and TAM variables—behavioral attitudes and intention to use drones. Only H4a regarding the link between risks and behavioral attitudes toward using drones was supported. In contrast to the prediction in H4b, the effect of drone-related risks on intention to use them was not significant. However, a significant indirect path was found to exist between risks and intention to use, mediated by behavioral attitudes.

## Discussion

In this paper we examined individuals' intentions to use drones and how they engaged with the benefits, risks, and uses of drones during a PHE in Israel. The results of the empirical analysis provide strong support for most of the hypotheses. In what follows, we discuss the findings of the study from both a theoretical and practical perspective.

### Theoretical implications and discussion

From a theoretical perspective, we integrated the extended TAM and TTF theory, as well as social motivation and perceived risks, to explore public drone acceptance and utilization in the context of a PHE. This study advances the existing knowledge about the TAM and TTF in several ways. First, an integrated framework combining the two models and the addition of social motivation and perceived risks, which was developed on the basis of previous studies in different technological contexts (39–41), was presented and empirically confirmed in the new context of drone technology. Second, the model facilitates an in-depth understanding of the mechanisms of TAM and TTF separately, as well as of those that combine them, which underlie drone adoption in the unique context of PHE. Further implications and discussions for each model and the additional constructs are described in the reminder of this section.

In regard to the TAM, the findings indicate that perceived ease of use is not a predictor of perceived usefulness. This finding contradicts the original assumption underlying the TAM model but aligns with the previous findings of a study that explored drone acceptance in agriculture. The researchers attributed this finding to the multiplicity of uses entailing different skill levels; in other words, the effect of perceived ease of use is different for each application and cannot point to a clear direction (73). Our finding supports this notion. Additionally, some of the drone uses in a PHE, such as performing surveillance tasks, are operated remotely by organizations and not by the users themselves and, as such, perceived ease of use may be less relevant in these regards. Future studies should distinguish between uses that require interaction with the public and those that do not.

We also demonstrated in this study that the effect of behavioral attitudes on intention to use drones is both significant and positive, and that behavioral attitudes are an important mediator between perceived ease of use and perceived usefulness (on the one hand) and intention to use drones (on the other), as theorized in the original model of technology acceptance (74). In addition, perceived ease of use also had a direct effect on intention to use drones. These findings align with findings from previous studies [e.g., (41, 75)] and point to perceived ease of use as an important factor in shaping public attitudes as well as acceptance of drones. Perceived ease of use expresses aspects related to human-technology interaction (76) and, in this case, of human-drone interaction (77). Our results suggest that the interaction characteristics are a powerful aspect of drone acceptance, and further research should focus on improving this interaction in the context of drone use in PHEs.

Additionally, our results suggest that extending the TAM through the integration of TTF, social motivation, and perceived risks yields greater explanatory power of the intention to use drones than the TAM can provide alone, as also reported by a recent study that explored customers' adoption of drone food delivery services (78). However, the context of food delivery is very different from the one explored in the current study, and thus, additional studies are required to further support this notion.

The TTF by itself was also found in the current study to be a robust model in which task and technology characteristics significantly determine the intention to use drones in PHEs. Furthermore, the results suggest that task-technology fit can influence both core components of the TAM, perceived ease of use and perceived usefulness, and that individual-technology fit influences perceived ease of use, thus contributing to drone acceptance on more than one path. As speculated, drone features that support the maintenance of health during a PHE (i.e., task-technology fit) can shape individuals' positive perceptions regarding both ease of use and usefulness of drones. The belief of a person that they can independently interact with a drone (i.e., individual-technology fit) is an antecedent for perceived ease of use. This finding further supports the importance of human-drone interaction in the current context as stressed above. These results align with those of a prior study which indicated the effect of the TTF on perceived ease of use and perceived usefulness of gamification in higher education (39). In sum, the better the fit between the individual, the task, and the technology, the greater the likelihood of drones being perceived positively. The results of this study can serve as a reference for future studies on drones with the TTF model.

The proposed model extends the role of social motivation in regard to drones by integrating two factors: social recognition and social influence. As predicted, social recognition was found to influence perceived usefulness and to have a direct effect on the intention to use them. Thus, a recognition of the benefits of drones by the healthcare system (a highly trusted institution in Israel), as well as positive public views regarding these benefits, seem to create a supportive environment for enhancing the promotion of drone public acceptance. These results align with those of previous studies that explored social recognition as an external factor to the TAM (40, 55).

Social influence was found to have a strong effect on behavioral attitudes toward drones. This finding is not surprising given that peoples' beliefs about innovative technologies are well established as important factors shaping personal attitudes about these technologies (60, 79, 80). Together with the above, this finding further supports the argument regarding the importance of social motivation in the process of technology adoption.

Perceived risks were found to negatively affect behavioral attitudes toward drone use in PHEs. In addition, behavioral attitudes served as a significant mediating variable between perceived risks and intention to use drones. These findings correspond with those of previous studies both related to drone use (27, 49, 63) and to other technological [e.g., (61)], and emergency context (81–83). The results are consistent with the idea that perceived risks counterbalance individuals' propensity for using technologies potentially causing harm in various ways. Healthcare institutions wishing to introduce drones for use in PHEs will need to take this issue into consideration, as well as map out the perceived risks among different population groups.

In sum, the study findings enhance the understanding of factors impacting people's intention to use drones in the context of PHEs. The findings also broaden our understanding of user psychology and behavior, which has been mainly studied through a technology acceptance approach.

### Practical implications

From a practical perspective, the current study has unveiled several implications for drone companies as well as for healthcare organizations that wish to incorporate drones into their activity.

First, the centrality of perceived ease of use to the intention to use drones in PHEs suggests that drone companies, as well as healthcare organizations, should carefully design emergency drones and user interfaces to provide a friendly and intuitive experience in order to support acceptance by the general public. The study of human-drone interaction is relatively new, but previous findings show that designing interactions with drones to resemble interactions with pets can support their incorporation into daily life (77, 84). In the context of PHEs, further study is needed in order to understand whether interactions with drones in potentially stressful emergency situations can be facilitated by designing them to resemble interactions with healthcare practitioners, similar to interactions in telemedicine services.

Second, as the TTF factors and social recognition determine direct intention to use, as well as perceived usefulness and ease of use, a special emphasis should be placed on increasing awareness and clarifying the benefits of drones in maintaining health in general, and during PHEs in particular, factors that may not be intuitive or fully recognized by the public. Highlighting benefits such as expediting the receipt of emergency medical attention by using drones equipped with video cameras connected to health practitioners, or hastening medical supply replenishment by using delivery drones without having to leave home or another protected space, *via* public campaigns, may support drone utilization in PHEs. As the effects of social influence and of perceived risks on behavioral attitudes toward drones were found to be relatively high, we would recommend further exploring, characterizing, and mapping these factors among various population groups. Doing so might yield insights regarding specific populations who may hold negative beliefs or fear the risks of drones to a higher extent than others. Such insights can facilitate tailor-made campaigns and even a culturally-sensitive drone design to support drone acceptance across diverse populations.

### Limitations and future research

The current study had several limitations. First, its cross-sectional design precludes the determination of causality between the constructs examined, and in relation to the outcome variable of intention to use drones in PHEs. As user behavior is dynamic by nature, future studies should employ a longitudinal design that will enable an in-depth investigation of the interlinks between the factors involved in technology acceptance, as well as track trajectories of change specific to drone acceptance. Second, as only 48.5% of the variance in the intention to use drones in PHEs was explained, additional studies should incorporate other factors that may be relevant in this regard but were beyond the scope of the current analysis. Further, another study limitation may stem from the fact that we relied on a general sample of the population in Israel and did not control for factors such as ethnicity or cultural affiliation. As localizing user experience through culturally-sensitive designs has been recognized as an important factor for fostering effective communication and sustainable technological development (85, 86), future research should allow for subgroup analyses in a given population. For example, in the Israeli context, the Ultraorthodox Jewish population and the Arab population are two groups that should be explored in comparison with the general Jewish Israeli majority.

### Conclusions

Drones are becoming increasingly ubiquitous. Although many applications such as drone use for delivery, or for targeted search and rescue missions, have been discussed and even tested to a relatively large extent, the use of drones as part of response efforts to large-scale PHEs has led to more interest lately, due to the COVID-19 pandemic. Generally, there has been little examination of the factors involved in drone acceptance and even less in the context of PHEs. The integrated model that was developed and tested in the current study provides a robust and comprehensive framework to perform an in-depth investigation of the factors and mechanisms affecting drone acceptance in PHE. Furthermore, the empirical findings not only expand and deepen the existing body of knowledge but also provide valuable insights for public health researchers and practitioners, as well as others involved in emergency health management. The findings point to the centrality of factors related to human-drone interaction—such as perceived ease of use and individual-technology fit—in the process of drone acceptance and suggest that special attention should be provided to improving this interaction in the context of PHE. Additional factors such attitudes, task-technology fit, and social influence were also found to directly and indirectly influence the intention to use drones, and this further highlights the need to raise awareness of drone benefits in maintaining health during both routine and emergency times. As public opinion and belief regarding drones are still forming, the current findings will have an important impact on promoting drone public acceptance in PHEs and in general.

## Data availability statement

The raw data supporting the conclusions of this article will be made available by the authors, without undue reservation.

## Ethics statement

The study was reviewed and approved by the Ben-Gurion University of the Negev—Human Subjects Research Committee. The participants provided their written informed consent to participate in this study.

## Author contributions

SS: conceptualized, designed, analyzed, and wrote the original draft. JC: helped to design the study and edit the manuscript. Both authors contributed to the article and approved the submitted version.

## Funding

This work was supported by the BGU President Fund for COVID-19 Task Force.

## Conflict of interest

The authors declare that the research was conducted in the absence of any commercial or financial relationships that could be construed as a potential conflict of interest.

## Publisher's note

All claims expressed in this article are solely those of the authors and do not necessarily represent those of their affiliated organizations, or those of the publisher, the editors and the reviewers. Any product that may be evaluated in this article, or claim that may be made by its manufacturer, is not guaranteed or endorsed by the publisher.
