# Supplementary material for: Integrating drones in response to public health emergencies: A combined framework to explore technology acceptance
Source: Front Public Health. 2022 Oct 28;10:1019626. doi: 10.3389/fpubh.2022.1019626 (PMC9650287; doi:10.3389/fpubh.2022.1019626)
Supplement: Supplementary file 1 [file Table_1.DOCX]

Supplementary Material

Appendix A. Survey items

| Constructs | Items | Measures |
| --- | --- | --- |
| Perceived usefulness in PHEs (COVID-19) |  |  |
| I believe that during the COVID-19 pandemic drones can assist people by: | PU1 | Delivering supplies (e.g., food and water, portable power sources, etc.) |
|  | PU2 | Delivering medications |
|  | PU3 | Monitoring the location of people |
|  | PU4 | Reminders to take medications |
|  | PU5 | Reminders for other things (e.g., wearing masks when outside) |
|  | PU6 | Performing vital signs assessment (e.g., blood pressure, body temperature) |
|  | PU7 | Assisting in contacting medical practitioners by phone call or video call |
|  | PU8 | Assessing physical or mental distress and alerting doctor or nurse |
| Perceived ease of use | PEOU1 | Learning to interact with a drone would NOT be easy for me |
|  | PEOU2 | I would find it easy to get supplies of other shipments from a drone |
|  | PEOU3 | My interaction with a drone would be clear and understandable |
|  | PEOU4 | It would not be easy for me to become skillful in drone technology |
|  | PEOU5 | It would be easy for me to use a drone |
| Attitudes toward using drone in a PHE (COVID-19) | ATT1 | Using drones when responding to an emergency such as the COVID-19 pandemic is a good idea |
|  | ATT2 | Using drones when providing care during an emergency such as the COVID-19 pandemic is not a good idea |
|  | ATT3 | Using drones when responding to an emergency such as the COVID-19 pandemic could be beneficial for me and my family |
| Intention to use drone in a PHE (COVID-19) | ITU1 | I intend to use drones during an emergency such as the COVID-19 pandemic when it becomes available in my HMO |
|  | ITU2 | I intend to use drones during an emergency such as the COVID-19 pandemic as often as needed |
|  | ITU3 | I intend NOT to use drones during emergencies |
|  | ITU4 | To the extent possible, I would use drones to get different things (medical or non-medical) |
| Task-technology fit | TTF1 | Drones can help me fulfil my medical needs during an emergency such as the COVID-19 pandemic |
|  | TTF2 | Using drones fits with my healthcare practices |
|  | TTF3 | Drones are suitable for helping me maintain my health during an emergency such as the COVID-19 pandemic |
| Individual-technology fit | ITF | I can independently interact with a drone |
| Social recognition | SR1 | It is important for drones to be adopted by healthcare services |
|  | SR2 | It is important for drones to be accepted and trusted by others |
| Social influence | SI1 | Other people’s beliefs about drones encourage me to use them |
|  | SI2 | Other people’s beliefs about drones influence my degree of trust in them |
|  | SI3 | Other people’s beliefs about drones condition me to use them |
| Perceived risks | RISK1 | Using drones when responding to an emergency such as the COVID-19 pandemic can cause physical harm |
|  | RISK2 | Using drones when responding to an emergency such as the COVID-19 pandemic can violate my or others’ privacy |
|  | RISK3 | Using drones when responding to an emergency such as the COVID-19 pandemic can cause a breach in medical confidentiality |
